# Editorial: Cognitive and Brain Aging: Interventions to Promote Well-Being in Old Age

**DOI:** 10.3389/fnagi.2019.00353

**Published:** 2020-01-14

**Authors:** Carryl L. Baldwin, Pamela M. Greenwood

**Affiliations:** ^1^Department of Psychology, Wichita State University, Wichita, KS, United States; ^2^Department of Psychology, George Mason University, Fairfax, VA, United States

**Keywords:** cognitive aging, brain plasticity, cognitive training, physical exercise, multidomain interventions

This Research Topic is dedicated to the memory of Raja Parasuraman who passed away prematurely on March 22, 2015, ending a remarkable career spanning both diverse and intersecting research areas. Over the course of his career Raja used a variety of techniques ranging from behavioral studies, signal detection theory, electrophysiology, fMRI, and genetic analysis to contribute to different disciplines including human factors, cognitive neuroscience, and the intersection of the two in his founding of the field of neuroergonomics. He maintained an interest in cognitive aging throughout most of his career, studying the effects of not only disease states, such as Alzheimer's, but also interindividual differences in cognitive performance through cognitive genetics and cognitive training. This Research Topic celebrates the aspects of Raja's contributions that are related to cognitive aging and brain aging. Some of Raja's contributions in this area include work examining age-related changes in visuospatial processing and working memory, the role of the genetics of apolipoprotein E (APOE) in cognitive aging, in particular in Alzheimer's disease, and methods for supporting healthy cognitive aging. This work culminated in a book co-authored with Pam Greenwood entitled, “*Nurturing the Older Brain and Mind*” published by MIT Press (Greenwood and Parasuraman, 2012). Considered together, the contributions to this special issue build on Raja's work and, importantly, show a way for the field to move forward in the future.

Interventions are needed to ameliorate age-related cognitive decline which is a risk factor for the devastation of dementia, robbing older people of their well-being and shortening their lives. Interventions aimed at cognitive decline are the focus of this Research Topic dedicated to Raja Parasuraman. In recent years, cognitive aging research has pivoted from simply cataloging age-related cognitive decline to seeking interventions aimed at slowing or delaying that decline and improving quality of life in old age. Efforts to develop interventions to ameliorate cognitive aging rely on assumptions that interventions can heighten brain integrity and/or induce compensation for age-related decline in brain integrity. These assumptions are supported by evidence that aerobic exercise increases (a) adult-onset birth and integration of new neurons (neurogenesis) (Anacker and Hen, 2017; Kempermann, 2019) and (b) hippocampal blood flow in a manner related to improved recall (Pereira et al., 2007; Maass et al., 2015). These assumptions are also supported by evidence of cortical reorganization in older people following cognitive training (Maguire et al., 2000; Taub et al., 2002; Greenwood, 2007). This evidence of cortical reorganization related to training raises questions about the ability of the brain respond adaptively to age-related declines.

## Can the Aging Brain Compensate for Declining Integrity?

One of the important questions in cognitive training is whether the aging mind and brain exhibit active compensatory processes capable of countering cognitive decline. Two papers in this Research Topic address this question. Jiang X. et al. used a novel fMRI analysis method (local regional heterogeneity analysis) that showed individual differences in neuronal specificity were related to individual differences in task performance in a region-specific manner. They found that better neuronal specificity is associated with better task performance. Those findings are consistent with Baltes' notion that aging is not accompanied by compensation, but rather by neural “de-differentiation” accompanied by noise in information processing (Baltes, 1997). Also observing evidence refuting the existence of compensation, Harwood et al. in a cerebral blood flow study found that healthy older people showed greater left than right hemisphere activation during a vigilance task but still performed at a significantly lower level of vigilance compared to young adults. Alongside this evidence that older brains are not intrinsically able to compensate for declining brain integrity, is evidence that aerobic exercise interventions can improve brain integrity (Pereira et al., 2007; Maass et al., 2015).

## Age-Related Cognitive Decline Is Related to Changes in Brain Networks

Although the aging brain does not appear to compensate, *per se*, it does appear capable of reorganizing functionally. We previously argued that during aging the brain undergoes change in cortical representation through changes in processing strategy (Greenwood, 2007). Consistent with that view, several contributors to this topic found that age-related decline in executive function is related to changes in intrinsic brain networks. Several of the interventions considered in this topic are aimed at modulating intrinsic brain networks (Passow et al.; Reis et al., discussed in the next section). Avelar-Pereira et al. found that in both young and older adults the frontal parietal control network (FPN), thought to play a role in cognitive control and flexible switching from internal thoughts to external stimuli, was more closely coupled with the default mode network (DMN) at rest and more closely coupled with the dorsal anterior network (DAN) during task performance. Although the FPN-DMN connectivity was reduced in older adults under both conditions, the connectivity patterns between the FPN-DAN during task performance were similar in young and older adults. Interestingly, Avelar-Pereira et al. also found that reduced cerebral blood flow (CBF) in the DMN predicted the amount of DMN-DAN anticorrelation during task performance. Further, the observed anticorrelation was associated with better behavioral performance. Thus, Avelar-Pereira et al. found that human cerebral blood flow affected the integrity of brain function, consistent with previous human and animal work on aerobic exercise and brain plasticity (Pereira et al., 2007; Maass et al., 2015).

Callaghan et al. presented confirming behavioral evidence of age-related deficits in cognitive control. They compared age groups across the adult lifespan and found that ability to switch between spatial and temporal attention undergoes a decline starting in midlife. Methqal et al. examined age-related changes in executive control in a semantic association and rule switching task using behavioral performance and fMRI. Results provide further support for greater age-related changes in high level executive control tasks (e.g., like those that involve task switching) relative to more continuous task performance. Results of Methqal et al. also indicate that the shift in age-related activation from frontal to parietal regions can be viewed as a form of neurofunctional reorganization.

## Interventions

### Electrophysiological Interventions

That large-scale intrinsic brain networks undergo reorganization late in life suggests that electrophysiological interventions might have a normalizing effect. Two papers in this Research Topic do find benefits of such interventions on working memory: non-invasive brain stimulation (Passow et al.) and neurofeedback training to heighten certain EEG spectra (Reis et al.; Jiang Y. et al.) and to heighten attention and working memory (Jiang Y. et al.). Passow et al. argued that aging is accompanied by deficient neuronal gain control due to reductions in signal-to-noise within and between cortical networks. They reviewed evidence that non-invasive brain stimulation in the form of transcranial direct current stimulation (tDCS) alters patterns of brain electrophysiology in older people. They argued that anodal tDCS combined with cognitive training can improve cognition in older people by increasing functional connectivity in the fronto-striatal-parietal circuitry. Reis et al. took another approach to training brain electrophysiology. They found that an intensive alpha and theta neurofeedback protocol improved working memory (n-back) performance of older healthy people. Both of these studies are in the same vein as a recent high-profile demonstration that a form of non-invasive brain stimulation similar to tDCS can synchronize neuronal firing between prefrontal and temporal cortex with short-term benefits for working memory in older people (Reinhart and Nguyen, 2019). Overall, these non-invasive electrophysiological methods have considerable promise for improving working memory in healthy older people.

### Cognitive Interventions

Cognitive interventions have long been used as a means to improve cognition in older people. One of the fundamental questions in the cognitive training literature concerns whether training transfers to untrained domains and abilities—termed “far transfer” (reviewed in Greenwood and Parasuraman, 2016). Far transfer of training is considered by many researchers to be the most important goal of cognitive training. In the sizeable working memory training literature, far transfer is usually assessed in fluid ability (Gf), most commonly in Raven's Progressive Matrices scores (e.g., Au et al., 2014). A number of reviews and meta-analyses of cognitive training have been conducted, with results showing mainly small to medium effect sizes of far transfer (Simons et al., 2016), including in older people (Karbach and Verhaeghen, 2014; Melby-Lervag et al., 2016). The papers in this Research Topic are consistent with that literature. Payne and Stine-Morrow found that 3 weeks of home-based working memory training induced both near transfer and far transfer to various language functions. Souders et al. tested a large older sample using an active control condition and found that a “gamified” puzzle training intervention induced some near transfer but no far transfer. Similarly, Grönholm-Nyman et al. also found significant near transfer, but not far transfer from set shifting training in older people. Ware et al. did not find transfer from a language learning program. The finding of weaker or no evidence of far transfer is consistent with meta-analyses of working memory training in older people finding larger effect sizes for near transfer than far transfer (Karbach and Verhaeghen, 2014). Considered together with other evidence that cognitive training can have very durable effects (e.g., Willis et al., 2006; Anguera et al., 2013), this suggests that intervention research could also focus on durable near transfer. Although relatively neglected in the cognitive training literature, near transfer could help dementia patients learn and retain skills needed for daily tasks (de Werd et al., 2013).

### Mindfulness Interventions

Several of the contributors to this topic recognized the need to promote well-being in older people in addition to improving their cognition. Mindfulness training with the goal of promoting sustained attention in the context of “non-reactivity and acceptance” (Kabat-Zinn, 1990) has long been advocated as a tool to promote mental health. Two studies in this special topic examined mindfulness in older people. Fountain-Zaragoza and Prakash reviewed the topics of mindfulness disposition (trait mindfulness) and mindfulness training (mindfulness experience), pointing out both findings and weaknesses in the existing literature. Weaknesses include the need for standardized training protocols, including the need to assess transfer of training more broadly to include everyday function. Banducci et al. showed that older people exhibited improved their episodic memory recall after 4 weeks of the novel intervention of “active experiencing,” a tool used by actors that involves mindfulness. Although there have been previous studies using an active experiencing intervention, Banducci et al. used a rigorous intent-to-treat analyses and a non-imputation approach to missing data. Overall, these reviews show that mindfulness as an intervention has promise but is not yet supported by a body of rigorous investigation.

### Physical Exercise Interventions

As discussed previously in this editorial, this topic adds to the growing evidence that cognitive function in aging must be considered in the context of physical fitness. In observational studies, self-reported aerobic exercise level has been related to cognitive performance, gray matter volumes (Schultz et al., 2015), and AD biomarkers (Okonkwo and Kinsella, 1969). In this Research Topic, Thielen et al. extended that previous work to changes in both functional connectivity and inflammation, processes known to undergo age-related alteration. Higher compared to lower self-reported aerobic exercise was associated with increased encoding-related functional connectivity in a brain network linked to episodic memory (mPFC, thalamus, hippocampus precuneus, and insula). Further, based on evidence of a role for inflammation in Alzheimer's disease (McGeer and McGeer, 2001), systemic inflammation was assessed. Increased connectivity was related to decreased in interleukin 6, suggesting lower inflammation in those with higher self-reported aerobic exercise levels.

Burzynska et al. reported that an older group randomly assigned to learn complex social dance sequences showed that integrity of the fornix (the major output tract of the hippocampus) increased over 6 months. In contrast, fornix integrity decreased in an older group randomly assigned to walking, to walking plus nutrition, or to active control. Jonasson et al. also examined brain structure changes over a 6 month exercise intervention and found that only the group randomly assigned to aerobic exercise showed improved cognition (on a composite measure of episodic memory, processing speed, updating, and executive function) as well as showing an association between cognitive score and dorsolateral PFC cortical thickness. A group assigned to stretching and toning did not show those effects. The results of two contributors (Burzynska et al.; Jonasson et al.) are consistent with the above-discussed evidence of the importance of aerobic exercise for hippocampal blood flow and adult neurogenesis for cognition in old age (Pereira et al., 2007; Maass et al., 2015). Finally, Stillman et al. reviewed the complex relation between physical activity and obesity. Obesity is a risk factor for dementia, making the recent increase in adult obesity in the US to nearly 40% very concerning. Yet the relation between obesity and physical activity is complex. Exercise interventions alone are largely ineffective at reducing obesity. Benefits on neurocognitive function after an exercise intervention depend on cardiovascular fitness rather than on adiposity. Nevertheless, combined dietary and exercise interventions are effective in counteracting the negative effects of obesity by increasing metabolic function, decreasing inflammation, and increasing neural plasticity. Specifically, dietary interventions do improve neurocognitive function despite having no effect on cardiovascular health. There is increasing recognition of the importance of diet in aging brain health, a topic we will return to at the end.

### Design Issues in Intervention Research

Several design issues are important for development of effective interventions against cognitive aging. A problem that looms over all training interventions is the design of control conditions, an issue addressed by Motter et al.. Their review article discussed major factors to control for when developing an active control condition, namely expectancy, engagement, motivation, novelty, and therapist interaction. They then frame their discussion in terms of the desirable qualities of an active control condition, and how lack of attention to these factors has hindered interpretation of the results of training studies in many previous investigations.

Another design issue concerns the effort put forth by participants. It appears that for benefits from interventions to be realized, additional effort is needed by the participant. Ayasse et al. found that despite poorer hearing acuity in the older group, sentence comprehension was as rapid in an older as in a younger group. However, the older group expended greater effort as measured in the pupillary response. These findings fit co-author Wingfield's previous work on the decremental effects of age-related hearing impairments on cognition. The finding of Ayasse et al. on a compensatory effect of increased effort suggest a direction for future work explicating the role of increased effort in other training domains.

Another design issue for cognitive interventions is the length of training. Most cognitive training studies have trained over weeks only, but the well-known Advanced Cognitive Training for Independent and Vital Elderly (ACTIVE) study added periodic booster training found effects on cognitive function 5 years after reasoning training (Willis et al., 2006) and 10 years after reasoning and speed of processing training (Rebok et al., 2014). In this Research Topic, Requena et al. trained regularly for 6 years using techniques and strategies for improving working memory and mood in an everyday memory context. Although far transfer *per se* was not assessed, Requena et al. found effects on memory after 6 years, thereby partially confirming the findings of the ACTIVE study for reasoning and speed of processing training. However, the duration of the interval between training and testing used by Requena et al. was not clear. Also finding durable effects, Gronholm-Nyman et al. trained on set-shifting for only 5 weeks, but also found that benefits of training on memory were retained at the 1 year follow up test. Souders et al. also emphasized the need for additional long duration studies such as Requena et al. or at least assessment after a long delay. Requena et al. discuss potential benefits of using a booster training approach in light of the apparent success of that approach in the ACTIVE study. Considered together with the ACTIVE study, these studies in our Research Topic emphasize the benefits not only of cognitive training that is long-term, but also of longer-term assessment of effects of training regardless of training duration.

## Conclusions

Considered together, the papers on cognitive aging in this Research Topic dedicated to Raja Parasuraman suggest the field of cognitive aging is undergoing a “sea change, into something rich and strange” (The Tempest, Act I, scene 2, Shakespeare, 1610). Parasuraman, a lover of Shakespeare and of bringing diverse perspectives and scientific communities together to forge new conceptual ground, would be pleased with the spirit and direction of this special issue.

Although cognitive aging research groups have typically each focused on only one type of intervention (cognition or diet or exercise), there may be synergism in combined interventions. A recent large scale study found that cognitive decline in aging can be slowed by a 2-year intervention of combined aerobic exercise, specific cognitive training and social stimulation, plus adoption of a Mediterranean diet. Participants were low-cognitive functioning but non-demented older people (Ngandu et al., 2015). Similar results from multidomain interventions have been reported in older people showing cognitive decline (Schelke et al., 2018) and in patients with “mild cognitive impairment” (MCI), a precursor state to Alzheimer's disease (Rovner et al., 2018). This evidence is consistent with Stillman et al. in showing the interrelatedness of diet and physical fitness on neurocognitive health. Despite a number of failed clinical trials on specific vitamins and supplements on cognitive aging (e.g., vitamin E, fish oil, etc.), the complex Mediterranean diet (involving high consumption of vegetables, legumes, fruits and nuts, cereals, olive oil and fish, moderate consumption of ethanol, and low consumption of red meat) appears to be effective, with greater adherence to the diet yielding greater cognitive benefits (Féart et al., 2009; Martínez-Lapiscina et al., 2013). The effectiveness of these multi-domain interventions points to the previously-mentioned importance of cardiovascular health (e.g., blood flow) in supporting neural plasticity late in life (Pereira et al., 2007; Maass et al., 2015; Avelar-Pereira et al.). It also points to the relative paucity of theoretical and empirical work on underlying mechanisms of combined effects. Importantly, although it is early days, the effectiveness of these comprehensive interventions stands in contrast to the failure of drug trials aimed at Alzheimer's disease and Mild Cognitive Impairment (Servick, 2019). Importantly, there are now several ongoing large scale multi-dimensional trials (USA POINTER, Baker, 2018) modeled on the Finnish FINGER study (Ngandu et al., 2015).

This Research Topic raises another important question. If cognitive interventions induce far transfer with small effect sizes but induce near transfer with medium to large effect sizes, perhaps cognitive interventions should also focus on durable near transfer. Rather than aiming interventions at improving cognitive globally in far transfer, the focus could be on interventions that improve specific cognitive skills and abilities regardless of far transfer. Near transfer from updating training to working memory would be important for daily functioning in the face of cognitive aging. Such an approach is currently being used to help dementia patients learn and retain skills needed for daily tasks (de Werd et al., 2013).

To summarize the main themes of this Research Topic, it appears that while the aging brain cannot compensate for declining integrity (Jiang X. et al.; Harwood et al.), brain and cognition in older people can benefit from aerobic exercise (Burzynska et al.; Jonasson et al.; Thielen et al.) and dietary interventions (Stillman et al.). Further, certain types of cognitive training have been shown to have very durable effects (Requena et al.; Grönholm-Nyman et al.) and also to more strongly induce near transfer than far transfer (Payne and Stine-Morrow; Grönholm-Nyman et al.). This points to durable near transfer as a useful aim for cognitive aging interventions. A recent innovation in the field shows that electrophysiological interventions have the potential to normalize large-scale networks in aging (Passow et al.; Reis et al.). Finally, the somewhat fragmented field of various cognitive and brain aging interventions may be unified by long-term multidomain interventions, the importance of which was emphasized by Stillman et al..

By its diverse contributions, this research topic reifies the sea change just beginning to transform research efforts to ameliorate cognitive and brain aging. The status quo can be characterized as separate research communities each focused narrowly on cognition, on exercise, or on diet. What is needed is collaboration across scientific fields to investigate possible synergisms involved in combined interventions involving cognitive training, aerobic and resistance exercise training, and diet. The Finnish FINGER and the USA POINTER studies are leading the way to an approach which allows investigation into possible synergisms. Such a change has the potential to forge new conceptual and theoretical ground in line with the spirit of this special issue.

## Author Contributions

PG and CB contributed equally to the writing of this editorial.

### Conflict of Interest

The authors declare that the research was conducted in the absence of any commercial or financial relationships that could be construed as a potential conflict of interest.
